# In vitro validation of the tear matrix metalloproteinase 9 in-situ immunoassay

**DOI:** 10.1038/s41598-020-71977-w

**Published:** 2020-09-15

**Authors:** Seung Pil Bang, Myeong Jin Son, Harim Kim, You Hyun Lee, Jong Hwa Jun

**Affiliations:** 1 Department of Ophthalmology, Keimyung University School of Medicine, 1035 Dalgubeol-daero, Dalseo-gu, Daegu, 42601 Republic of Korea; 2 Department of Biomedical Engineering, University of Rochester, Rochester, New York United States of America

**Keywords:** Diagnostic markers, Diagnostic markers

## Abstract

We aimed to validate a tear MMP-9 in-situ immunoassay (InflammaDry) and to identify factors that could affect results or interpretation. Three factors were examined: sample concentration, volume, and time. Recombinant human (rh) MMP-9 (10 or 20 μl; 0, 12.5, 25, 50, 100, 200, 500, and 1,000 ng/ml) was applied to the kit and the detection limit and assay reproducibility were examined. At a rhMMP-9 volume of 10 μl (≥ 50 ng/ml), all positive results were identified by densitometry at 10 and 20 min; however, after 20 min, more than half of the nine ophthalmologists interpreted a positive result. At a rhMMP-9 volume of 20 μl (≥ 25 ng/ml), ophthalmologists and densitometry identified almost all test lines at 10 and 20 min. At 10 μl, densitometry showed a linear dose–response pattern. At 20 μl, densitometry showed a linear dose–response pattern at concentrations up to 500 ng/ml; however, full saturation was achieved at concentrations ≥ 500 ng/ml. When the same amount of rhMMP-9 was applied, the density result increased significantly upon doubling of the solvent volume (i.e., by adding the same volume of PBS to a sample). InflammaDry showed a high inter- and intra-assay coefficient of variation at 10 min (28.4% and 24.7%, respectively). The results of the MMP-9 in-situ immunoassay varied significantly depending on sample volume. Therefore, when interpreting the results, careful attention must be paid to tear volume.

## Introduction

The recently renewed definition of dry eye disease by the Tear Film & Ocular Surface Society (TFOS) Dry Eye Workshop (DEWS) II emphasizes tear film instability, hyperosmolarity, and ocular surface inflammation and damage as triggers of a vicious cycle in those with dry eye^1,2^. In general, first-line diagnostic tools for dry eye include tear break-up time, the Schirmer test, symptom scoring questionnaires, and slit lamp biomicroscopy using fluorescein or lissamine green. However, these traditional diagnostic tools suffer from subjective characteristics, lack of specificity, and low reproducibility; therefore, other novel methods for diagnosing dry eye have been developed^3^. Although elevated tear osmolarity and ocular surface inflammation are important indicators of dry eye disease (DED), first-line methods do not reflect these pathophysiologic factors^4^.

Recently, two diagnostic modalities were introduced for objective evaluation of tear hyperosmolarity and matrix metalloproteinase 9 (MMP-9) levels (a measure of ocular surface inflammation)^5–7^. In the latter case, InflammaDry (Quidel Corporation, San Diego, CA, USA) is a disposable single-use immunoassay designed to detect active and latent forms human MMP-9 in tears^8^. The manufacturers state that the assay requires a tear volume of 10 μl to detect > 40 ng/ml of MMP-9 and is able diagnose dry eye within 10 min. MMP-9 participates in remodeling of the extracellular matrix after wounding of the corneal surface and is implicated in the pathogenesis of sterile corneal ulceration, ocular allergy, fungal keratitis, burns, advanced keratoconus with irregular surface, active pterygia, conjunctivochalasis, blepharitis, and dry eye^9–16^. In addition, MMP-9 levels in cases of dry eye or keratoconus are used to guide the decision to use anti-inflammatory treatments such as topical cyclosporine^9,17^. The MMP-9 immunoassay is a type of lateral flow immunoassay (LFIA). Therefore, InflammaDry has fundamental characteristics similar to those of LFIA. Koczula and Gallotta^18^ point out that LFIA, by its nature, has a marked impact on kit accuracy. Since LFIA is capable of both providing quantitative and quantitative results, it is important to determine whether InflammaDry generates quantitative results based on the concentration of MMP-9. From a clinical perspective, a fair number of clinicians wonder whether band density is a direct or indirect representation of severity of inflammation on the ocular surface. If InflammaDry can reflect tear concentrations of MMP-9 quantitatively, this may play a crucial role in determining the severity of inflammation and improve the outcomes for dry eye patients beyond a simple diagnosis. Furthermore, clinical interpretation of the faint test (red) line during examination can be ambiguous, particularly because conditions involving deficiency of aqueous tears (such as Sjögren syndrome^19^ or ocular graft-versus-host disease^20^) yield negative results despite severe signs and symptoms of dry eye. Therefore, we need to know exactly how the results of the kit change as tear volume changes. In addition, the kit instructions suggest that it is read initially after 10 min, and that a further 10 min should be allowed if the initial result is negative. Therefore, we need to know exactly how the kit's response changes over time. Also, conjunctival sac remnants in some eye drops may generate false-negative or false-positive results. Therefore, the present study aimed to validate the MMP-9 immunoassay by examining the effects of time, sample concentration, sample volume, and drug interaction on the results.

## Materials and methods

### Reagents

The MMP-9 immunoassay kit (InflammaDry) was purchased from Quidel Corporation (San Diego, CA, USA). The TearLab osmolarity system was purchased from TearLab Corporation (San Diego, CA, USA). Lot numbers used in the experiments were 1,801,207, 1,803,262, 1,804,133, 1,804,023, 805,149, and D613208 (in sample collectors), D631402, 1,805,241, 1,806,129 (in test cassettes), and 160634AA, 180148AA, 180270AA, 180232AA (in buffer vials). The active form of recombinant human MMP-9 (rhMMP-9, activated by 1 mM p-aminophenylmercuric acetate) was purchased from BioLegend^®^ (550502, San Diego, CA, USA). According to the manufacturer's manual, this recombinant protein (> 95% purity) originates from the supernatant of 293E cells and is expressed with a C-terminal 8His tag and a linker sequence. Serial dilution of 100 μg/ml rhMMP-9 with phosphate buffered saline (PBS) produced samples containing 12.5, 25, 50, 100, 200, 500, and 1,000 ng/ml of active rhMMP-9. Reference standard control solutions (100 ± 2, 290 ± 2, and 500 ± 2 mOsm/L) for osmolarity were purchased from Precision Systems Inc. (Natick, MA, USA).

### Experimental procedures

#### Assay protocol

**Figure 1:**
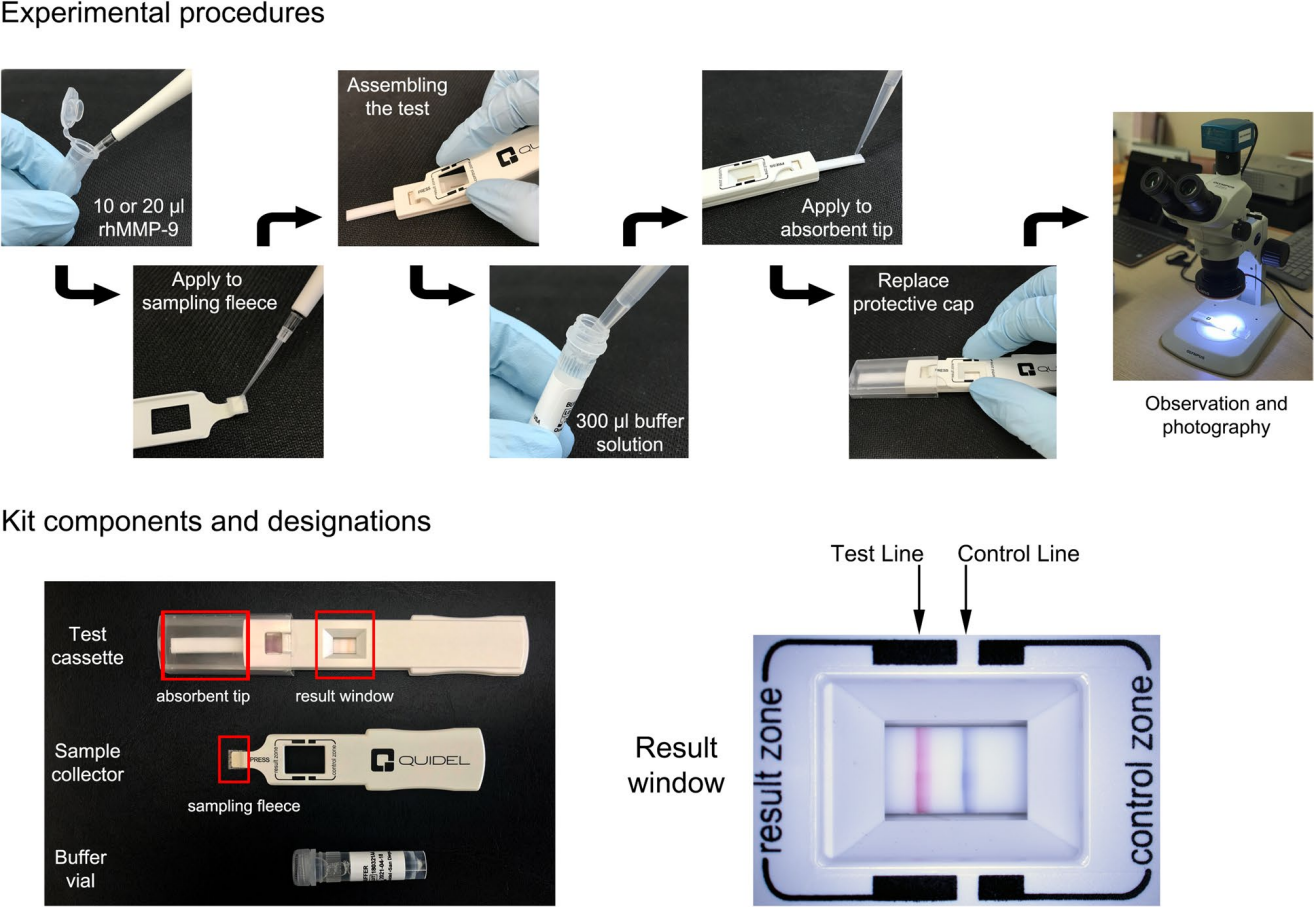
Scheme of assay protocol (top panel) and kit components and designations (bottom panel).

The effects of sample volume on the immunoassay results were tested using two sample volumes (10 and 20 μl), each containing 0, 12.5, 25, 50, 100, 200, 500, or 1,000 ng/ml of rhMMP-9. Samples were applied to the sampling fleece of the sample collector using a micropipette. Each sample concentration was obtained by twofold serial dilution of a stock solution (1,000 ng/ml) of rhMMP-9 in PBS. After assembling the sample collector and test cassette, 300 μl of buffer solution from the buffer vial was applied to the absorbent tip using a micropipette. The assay protocol is summarized in Fig. 1.

#### Time-dependency of the assay results

To examine the effect of time course on the immunoassay results, a stereomicroscope (SZ61TR, Olympus Corporation, Shinjuku, Tokyo, Japan) was used to take photographs automatically at 1 min intervals after completion of the assay protocol. Photographs were taken over a period of 20 min and images were analyzed using an iSolution Lite image analyzer (IMT iSolution Inc. Burnaby, BC, Canada). All experiments were repeated at least three times. Illuminance of both the laboratory and the microscope light source was measured with a light meter (TES1339 Light meter pro, TES Electronical Electronic Corporation, Taipei, Taiwan) At every time point, background illuminance was set to 400 lx, and the light source of the microscope was set to 65,000 lx.

#### Measurement of rhMMP-9 band density

Each image was evaluated using Image J^21^ (NIH, Bethesda, MA, USA) and interpreted by three well-trained observers (M.J.S., H.K., and Y.H.L.) using the same protocol. Captured images were converted to grayscale images and rotated 90° clockwise. Plots of band densitometry were trimmed and bound by the tangent closest to the radius of the curvature generated by the image background. The peak area of the test line was measured by each observer. Technical triplicate measurements from three different assays were averaged.

### Evaluation of detection limit

#### Detection limit evaluated by densitometry

The detection limit of the assay is defined as the lowest concentration that produces a positive test (red) line. The positivity of an assay was determined by detecting a peak on the test line densitometry plot.

#### Detection limit evaluated by the naked eye

This evaluation was conducted in accordance with the ethical principles of the Declaration of Helsinki. The study protocol and written informed consent were approved by the Keimyung University Medical Center Institutional Review Board (IRB no. DSMC 2017-06-008). To assess the detection limit using the naked eye, nine ophthalmologists examined a single-blind experiment. Briefly, volumes of 10 and 20 μl (each containing 25, 50, 100, and 200 ng/ml of rhMMP-9) were applied to the sampling fleece; each concentration was assayed in triplicate. All assembled assays were arranged in a random sequence and after 10 and 20 min all were interpreted simultaneously by nine ophthalmologists. The cut-off value of 50% positivity was based on the official Package Insert released by the manufacturer (https://rpsdetectors.com/en/wp-content/uploads/2014/03/SPEC-MKT-065.0-InflammaDry-CLIA-Waived-Package-Insert-US.pdf).

### Reproducibility

#### Inter-assay variability

The commercially available MMP-9 immunoassay can only test one sample per assay. Therefore, to evaluate the inter-assay variability, we measured the density of the test lines generated by 10 μl containing a high (500 ng/ml) concentration of sample and by 20 μl containing a low (200 ng/ml) concentration; both were incubated for 10 and 20 min. Tests were performed on six separate days using two separate batches (Lot number 1,804,023, sample collector; Lot number 1,805,241, test cassette, and Lot number 1,804,133, sample collector; Lot 1,805,241, test cassette). The 12 samples used in the 12 different assays were obtained from six separate aliquots of rhMMP-9. As assay reproducibility is vital to validate consistency of performance, the coefficient of variation (CV, %) was calculated as follows: standard deviation (SD)/mean × 100.

#### Intra-assay variability

Intra-assay variability was determined using the protocol described in 2.3.2.1 above, but only a single batch (Lot number 1,804,023, sample collector; Lot number 1,805,241, test cassette) was used and tested six times on the same day. The CV was also calculated, described as above.

#### Drug interactions

To investigate interactions between the MMP-9 immunoassay and components within eye drops, 10 μl of rhMMP-9 (200 ng/ml) was applied to the sample collector, followed by addition of 10 μl of eye drop sample. The following eye drops were used: Elazop (brinzolamide/timolol; Novartis, Basel, Switzerland), Alphagan-P (brimonidine tartrate 0.15%; Allergan, Dublin, Ireland), Isopto Carpine (pilocarpine hydrochloride 2%; Novartis), Bronuck (bromfenac sodium 0.1%; TaeJoon, Seoul, Korea), Cravit (levofloxacin 0.5%; Santen, Tokyo, Japan), Diquas (Diquafosol sodium 3%; Santen), Ikervis (cyclosporine A cationic emulsion 0.1%; Santen, Evry, France), Lotemax (loteprednol etabonate 0.5%; Bausch & Lomb, Rochester, NY, USA), Pazeo (olopatadine hydrochloride 0.7%; Novartis), Lastacaft (alcaftadine 0.25%; Allergan), Mydrin-P (tropicamide 0.5%/phenylephrine 0.5%; Santen), Mydriacyl (tropicamide 1%; Novartis), Cyclogyl (cyclopentolate HCl 1%; Norvatis), Isopto Atropine (atropine hydrochloride 1%; Novartis), and Refresh Tears (carboxymethylcellulose sodium 0.5%; Allergan). The test line was photographed after 10 and 20 min, the density was measured, and the results were compared with a control sample (200 ng/ml of rhMMP-9; 20 μl).

#### Effect of sample volume on TearLab osmometer detection

To analyze the effect of sample volume on the TearLab osmometer, 0.2, 0.5, 1.0, and 2.0 μl of standard reference solutions (290 and 350 mOsm/L) were applied to the TearLab osmometer test cards. This experiment was repeated five times for each reference solution.

#### Data handling and statistics

Statistical data were collected from individual experiments. The coefficient of determination, R^2^, was evaluated to identify a linear dose–response pattern. To assess the effects of addition of another 10 μl of PBS to the sample collector after application of the test sample, data were evaluated using an independent t-test. Statistical analyses were performed using SPSS version 12.0 (IBM, Chicago, IL, USA). A P value < 0.05 was considered significant.

## Results

### Effects of rhMMP-9 concentration on assay positivity and band density

#### Detection by the naked eye

**Figure 2:**
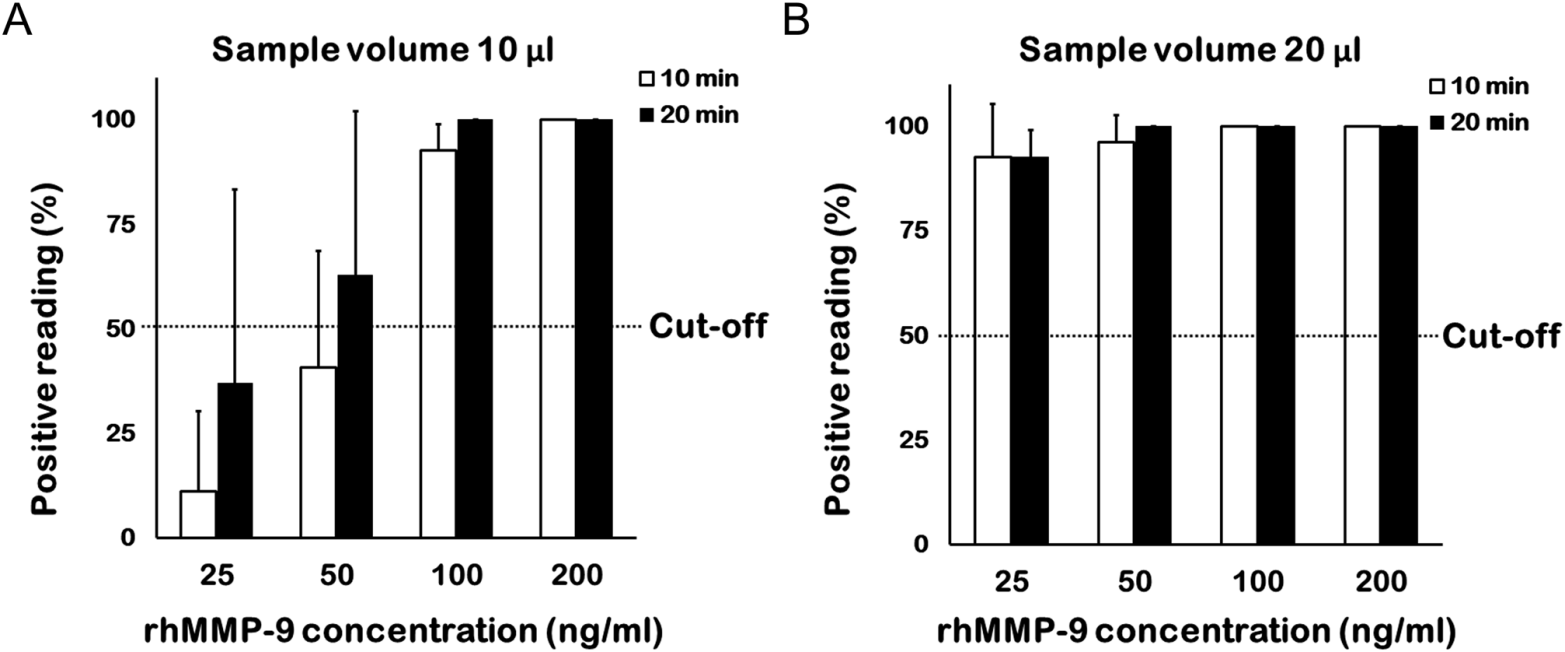
Percentage of ophthalmologists who identified a band positively at different concentrations. Nine ophthalmologists interpreted the MMP-9 immunoassay results. Each concentration comprised triplicate samples (total assays, n = 27). (**A**) At a sample volume of 10 µl, more than half of the ophthalmologists were able to detect a positive band at 50 ng/ml after 20 min. (**B**) At a sample volume of 20 µl, nearly all ophthalmologists detected a positive band at 25 ng/ml after 10 and 20 min.

When a 10 µl sample volume was tested, more than half of the nine ophthalmologists were able to identify a positive band at a concentration of 100 ng/ml after 10 min and at a concentration of 50 ng/ml after 20 min (Fig. 2A). When a 20 µl sample volume was tested, more than half of the nine ophthalmologists identified a positive band at 25 ng/ml after 10 min, and all identified a positive band after 20 min (Fig. 2B).

#### Detection limit and band density by Image J

**Figure 3:**
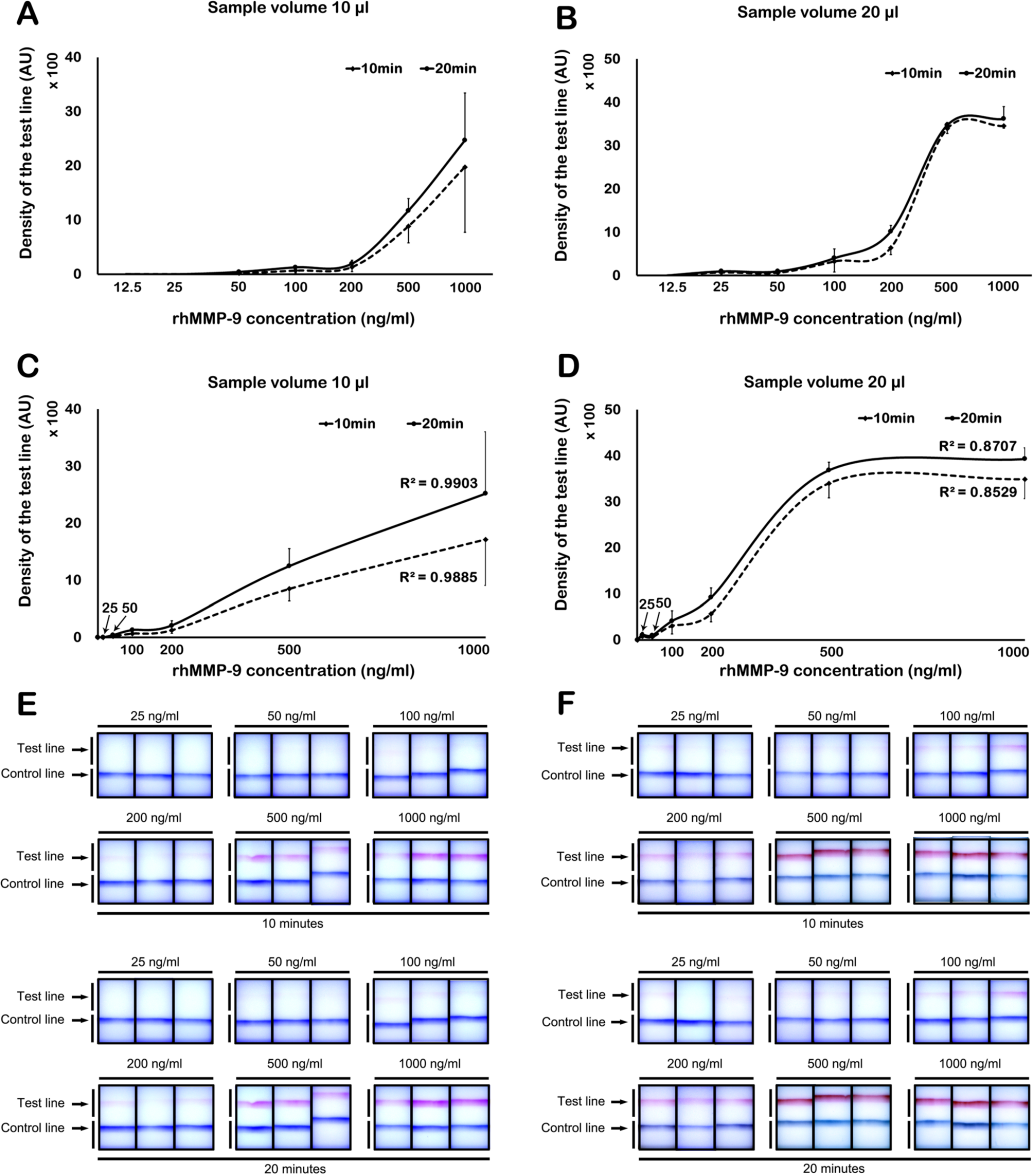
Concentration-band density relationship, as evaluated by densitometry. (**A**) Using 10 µl of rhMMP-9, a positive test line was detectable at concentrations ≥ 50 ng/ml. (**B**) Using 20 µl of rhMMP-9, a positive band was detectable at concentrations ≥ 25 ng/ml. (**C**) Using 10 µl of rhMMP-9, a linear dose–response relationship was evident. (**D**) Using 20 µl of rhMMP-9, a linear dose–response relationship was observed up to 500 ng/ml; however, the results reached a plateau above 500 ng/ml. (**E**,**F**) Visual appearance of the MMP-9 immunoassay at 10 and 20 min; visibility of the control and test lines at different concentrations of rhMMP-9 in 10 (**E**) or 20 (**F**) µl of sample. Data are expressed as the mean ± SD.

Image J identified a positive test line from a sample volume of 10 μl at a concentration of 50 ng/ml at both 10 and 20 min (Fig. 3A). The results showed a linear dose–response pattern (R^2^ = 0.9885 at 10 min; R^2^ = 0.9903 at 20 min; Fig. 3C). Using a 20 μl sample volume, a positive test line was detected at a concentration of 25 ng/ml at both 10 and 20 min (Fig. 3B). The density of test line showed a linear incremental pattern up to 500 ng/ml at both 10 and 20 min (R^2^ = 0.8529 at 10 min; R^2^ = 0.8707 at 20 min; Fig. 3D). At concentrations > 500 ng/ml, densitometry yielded a saturated signal (Fig. 3D). Figure 3E,F shows the visual appearance of the MMP-9 immunoassay at different concentrations of rhMMP-9 (in a sample volume of 10 or 20 µl) at 10 and 20 min, respectively.

#### Effects of sample volume on MMP-9 band density at different times

**Figure 4:**
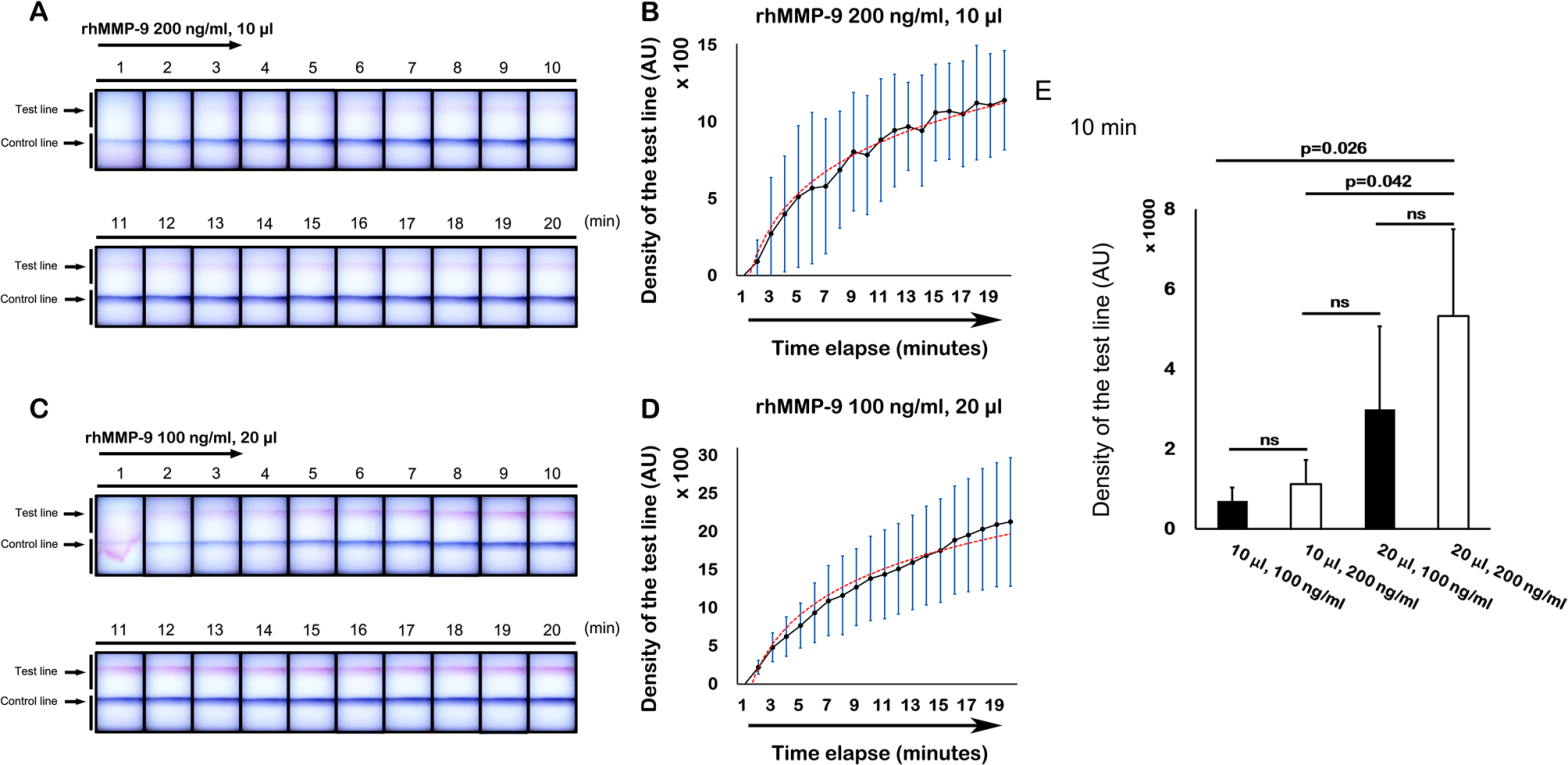
Time-dependent evaluation and volume-dependency of the MMP-9 immunoassay. (**A**,**B**) The band density was lower when the sample volume was smaller. (**C**,**D**) Although the same mass of solute was applied to the assay, the band density was higher when the sample volume was larger. (**B**,**D**) show gradual flattening of the curve up until 20 min, demonstrating a curve typical of a chemical reaction. (**E**) Relationship between rhMMP-9 concentration and sample volume. Data are expressed as the mean ± SD.

To elucidate the effects of total sample volume on band density over time, we compared the results of two individual experiments: 200 ng/ml rhMMP-9 in 10 μl and 100 ng/ml rhMMP-9 in 20 μl. Thus, the same amount of rhMMP-9 (0.2 ng) was applied to the sample collector, only the sample volume was different. Although same amount of solute was applied, the measured band density showed diverse results over time; density was higher when the sample volume was larger. The results for the 200 ng/ml (10 μl) sample showed an extremely faint band until 20 min (Fig. 4A). However, the results of the 100 ng/ml (20 μl) sample revealed a higher band density at 20 min; indeed, a very faint band was visible after 2 min (Fig. 4C). In addition, a graph of band density *versus* time showed a gradual flattening of the curve up until 20 min; such a curve is typical of a chemical reaction (Fig. 4B,D).

#### Effects of solvent volume on band density

**Figure 5:**
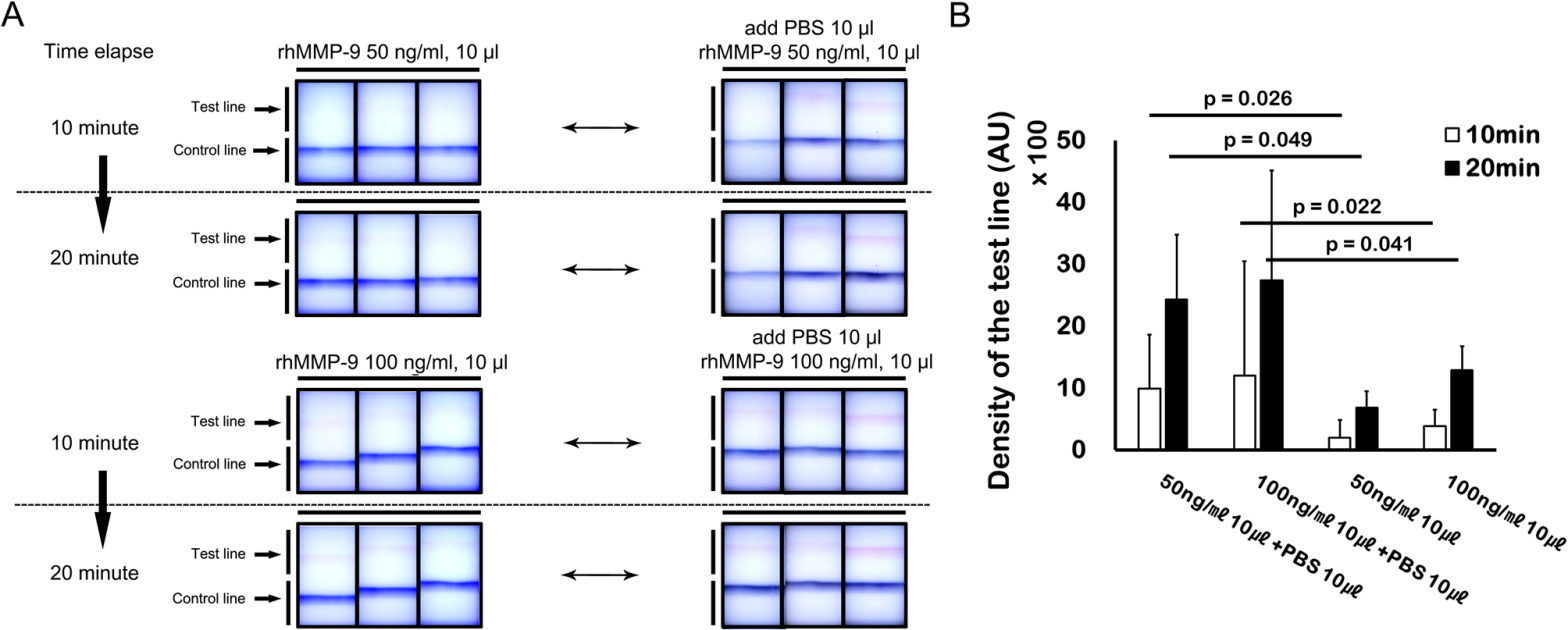
Additional experiments demonstrating the volume-dependency of the MMP-9 immunoassay. (**A**) Visual appearance of the MMP-9 immunoassay before and after addition of diluent (phosphate buffered saline, PBS). (**B**) Application of additional diluent after initial application of 10 µl of sample (50 or 100 ng/ml of rhMMP-9) to the sample fleece. Additional diluent (10 µl) increased the band density of 50 or 100 ng/ml of rhMMP-9 (n = 3). Data are expressed as the mean ± SD and evaluated using a paired t-test.

To evaluate the effects of solvent volume on band density, we conducted individual experiments using 50 ng/ml of rhMMP-9 in 10 μl and 100 ng/ml of rhMMP-9 in 10 μl; however, in these experiments, a further 10 μl of PBS were added to the sample collector. The results were compared with those obtained from experiments using 50 ng/ml (10 μl) of rhMMP-9 and 100 ng/ml (10 μl) of rhMMP-9, without further addition of 10 μl of PBS to the sample collector. Addition of 10 μl of PBS to the sample collector increased the band density of the 10 μl samples containing 50 and 100 ng/ml of rhMMP-9; these results were statistically significant (p < 0.05, paired t-test) (Fig. 5A,B).

#### Reproducibility of the MMP-9 immunoassay

**Figure 6:**
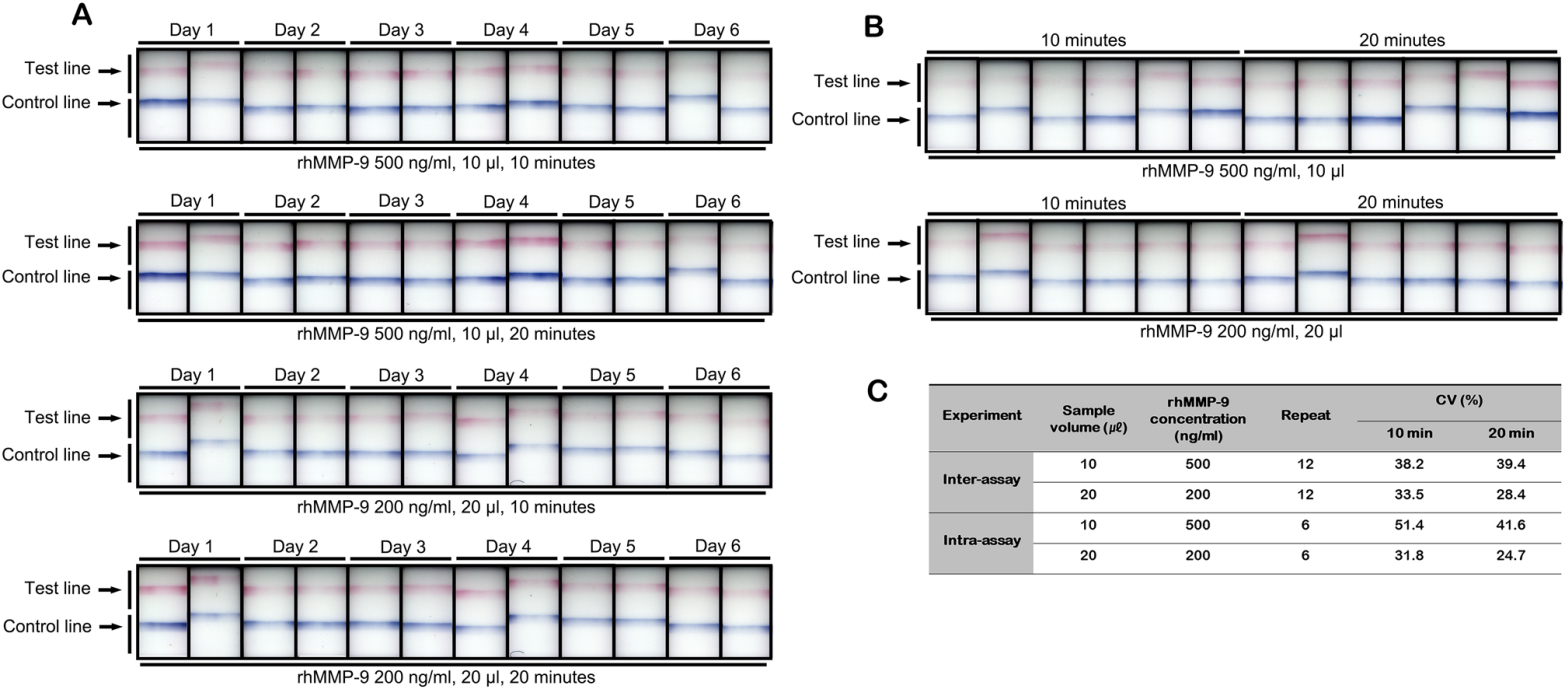
Visual appearance of the MMP-9 immunoassay used to assess inter-assay (**A**) and intra-assay (**B**) precision at different sample concentrations and volumes (10 µl, 500 ng/ml rhMMP-9; 20 µl, 200 ng/ml rhMMP-9). Calculated values are shown (**C**). CV = coefficient of variation.

Figure 6 presents a visual representation and the calculated parameters for assay repeatability. The calculated inter-assay CV value was 28.4% and 39.4%, while the intra-assay CV value was 24.7% and 51.4% at 10 and 20 min, respectively.

#### Effects of eye drops on band density

**Figure 7:**
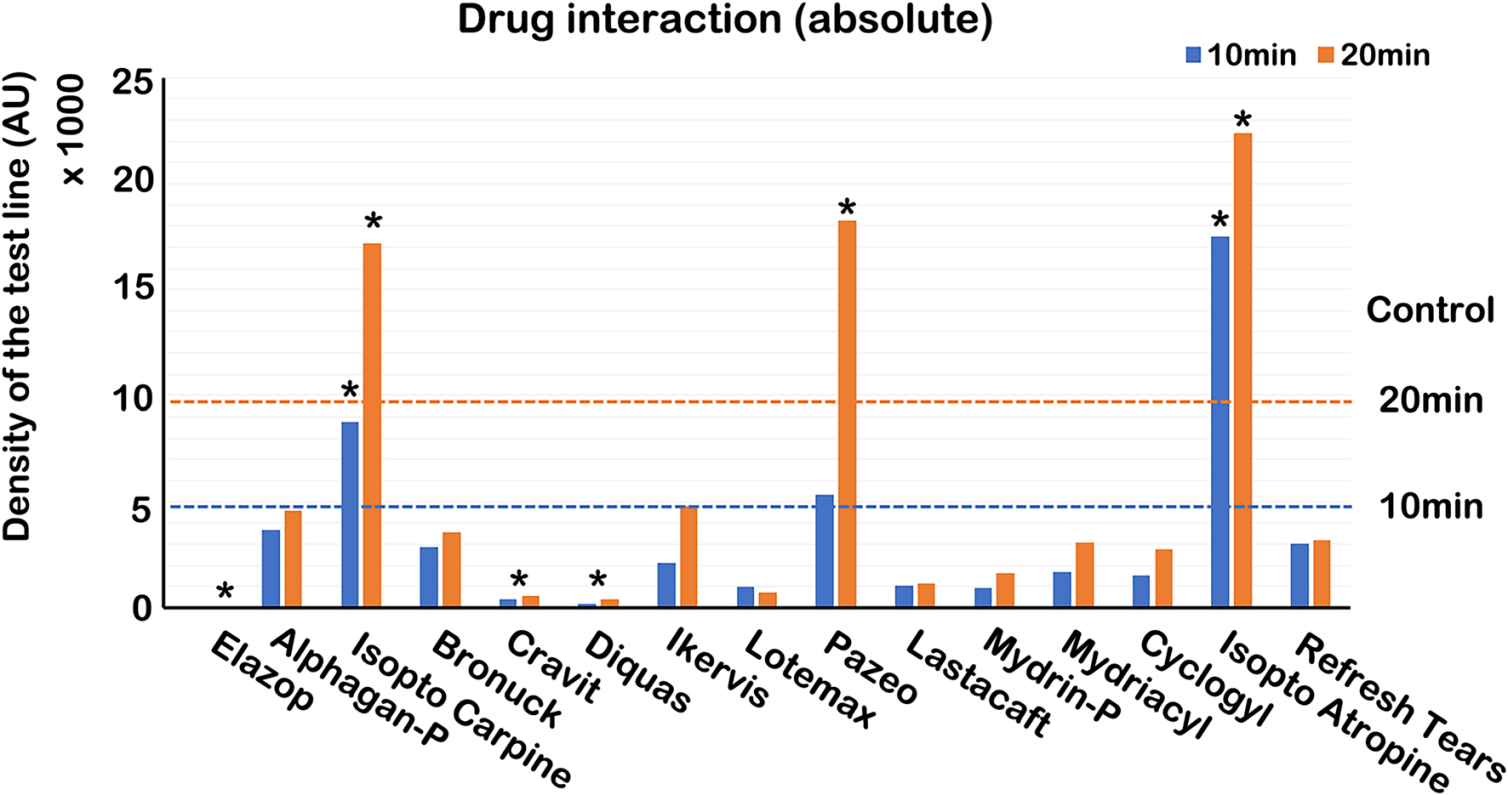
Effects of different eye drops on band density in the MMP-9 immunoassay. The density of the test line was evaluated after application of 10 μl (200 ng/ml) rhMMP-9, followed by 10 μl of each eye drop sample. The two dotted lines represent the average density of the control band (20 µl, 200 ng/ml rhMMP-9) after 10 and 20 min, respectively.

The band density of samples in the presence of Isopto Carpine, Pazeo, and Isopto Atropine was significantly higher than that of the control. However, band density in the presence of Elazop, Cravit, and Diquas was significantly lower than that of the control (Fig. 7).

#### Effects of sample volume on TearLab osmometer measurements

**Figure 8:**
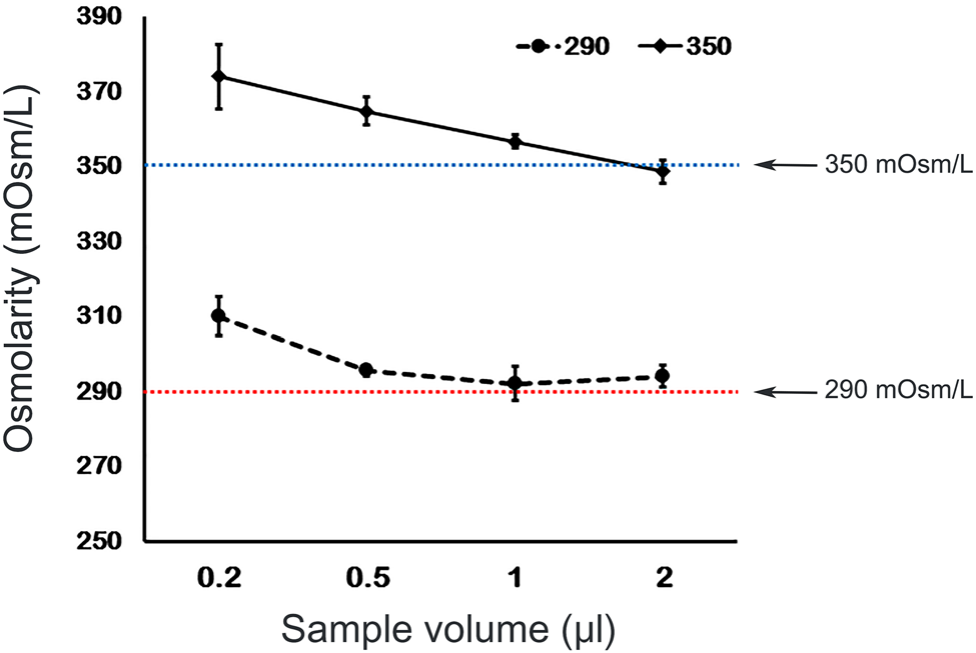
Effect of sample volume on TearLab osmometer measurements. Samples (0.2, 0.5, 1.0, and 2.0 μl) of two solutions (290 and 350 mOsm/L) were applied to the TearLab osmometer test card. The results for the 0.2 and 0.5 μl samples were significantly higher than the average osmolarity of the 290 and 350 mOsm/L standard solutions, respectively.

As the volume of the sample decreased, the measured osmolarity tended to increase gradually. At a volume of 0.2 μl, the measured osmolarity was higher (mean 24 ± 8.6 and 20 ± 5.3 mOsm/L) than the average for the 350 and 290 mOsm/L standard solutions, respectively (Fig. 8).

## Discussion

Over the past decades, a variety of diagnostic methods have been introduced to diagnose DED. Until recently, diagnosis and classification of DED depended only on symptoms and signs; however, symptoms and signs correlate poorly, and subjective evaluation is subject to bias^22,23^. Therefore, it necessary to establish standardized diagnostic methods for dry eye, as well as modalities for evaluating the therapeutic efficacy of drugs used for DED treatment^24^. For these reasons, tear hyperosmolarity and tear MMP-9 point-of-care (POC) tests based on pathological changes in dry eye have attracted attention. In addition, clinical modalities such as ocular surface interferometry to evaluate meibomian gland dysfunction, a major subtype of dry eye, are gaining traction^25^.

Recently, the tear MMP-9 in-situ immunoassay, InflammaDry, was introduced for qualitative diagnosis of DED. Although previous studies suggest that the assay provides accurate diagnosis for DED, in-situ diagnostics need to provide quality results from a limited tear volume^8,17,24,26,27^. DED is a multifactorial disease; as such, there are a wide variety of clinical features^22^. Sjögren syndrome, ocular graft-versus-host disease, and Steven-Johnson syndrome (all DED subtypes) result in markedly reduced tear secretion, making tear sampling challenging without additional manipulations^28–32^.

InflammaDry is based on a direct sampling micro-filtration method; as such, it is a kind of lateral flow immunoassay (LFIA). LFIA is a paper-based platform used to detect specific analytes in a specimen mixture. The sample is placed on a test strip and the results are displayed within 5–20 min^18^. The test is suitable for a variety of clinical samples, including urine, saliva, sweat, and blood^33–36^, and can detect specific antigens, antibodies, and gene amplification products^37–39^. When the sample is placed on the sample pad and comes into contact with conjugated detection antibodies, the latter combine with the target analyte and move to the test line in the running buffer. Bound target analytes are captured by anti-analyte antibodies at the test line^18^. This type of assay provides low-cost, rapid, simple, portable POC testing of liquid samples; however, low volume or the type of sample can affect the assay reliability or results^18^. The InflammaDry immunoassay is conducted by dabbing the collecting fleece 6–8 times against the lower palpebral conjunctiva, followed by pressing gently against the conjunctiva for an additional 5 s to saturate the fleece. Given that the lower tear meniscus volume is about 2.25 μl^40^, this instruction seems appropriate. After 10 min, primary interpretation can be made by confirming the positivity in the test line. If the clinician cannot identify the test line, the instruction manual recommends waiting an additional 5–10 min before proceeding with interpretation.

However, several questions arise. First, is there a concentration threshold for the band density that can be read as positive? Consistent and strong bands can be easily interpreted as positive, but very faint bands can be construed in several ways. In addition, does addition of solvent after tear collection generate a higher band density? Moreover, can band density be evaluated quantitatively using densitometry? If the assay provides reliable and reproducible quantitative information, ophthalmologists can try various anti-inflammatory treatments based on the assay results. Finally, does concurrent medication remaining in the conjunctival sac or tear affect the density of the test line?

Here, using a manufacturer-recommended cut-off value of 50%, we found that the detection limit by the naked eye was 100 ng/ml at 10 min and 50 ng/ml at 20 min (sample volume, 10 µl). These results are quite similar to those suggested by the manufacturer, i.e., a detection limit of 40 ng/ml. The kit instructions suggest that, initially, the result is read after 10 min; however, they also suggest allowing a further 10 min if the initial result is negative. Our observations suggest that these instructions are appropriate. However, with a sample volume of 20 µl, the detection limit by naked eye was 25 ng/ml at both 10 and 20 min. Theoretically, the volume of a tear sample can be 20 µl, so the results at 20 µl seem to detract from the reliability of the kit. Therefore, attention should be paid to interpretation of results in cases involving abnormalities of the lacrimal passage or punctal plugs, as tear volume in such cases may be higher than normal. Likewise, the detection limit by the naked eye in a sample volume of 10 and 20 µl was 50 and 25 ng/ml at both 10 and 20 min, respectively. These results indicate that sample volume affects the detection limit.

We also found that a sample with a different volume of solvent but the same amount of solute can give different results. We tested 10 µl of rhMMP-9 (200 ng/ml) and 20 µl of rhMMP-9 (100 ng/m) in the immunoassay. Since the total amount of analyte was same (2 ng), we expected that band density would be comparable. However, band density for 20 µl of rhMMP-9 (100 ng/ml) was nearly twice that of 10 µl of rhMMP-9 (200 ng/ml). Also, adding an extra 10 µl of PBS to the sample fleece after sample collection increased the intensity of the test line significantly. In order for the antigen–antibody reaction in the LFIA to occur evenly, the samples and their components should be distributed evenly in the sample fleece. However, if the volume of the sample is smaller than the amount needed for the assay, then the antigens are absorbed unevenly into the collecting pad of the sampling fleece. In this case, the efficiency of the antigen–antibody reaction is reduced, and the colorimetric reaction is weak. If the solvent is added to the sampling fleece, the MMP-9 in the sample is distributed evenly in the collecting pad of the sampling fleece, and the efficiency of the antigen–antibody reaction is increased, resulting in a strong colorimetric reaction^18^. These results suggest two things. One is that if the tear volume is very low then there is a high probability of negative or weak positive results, even if the concentration of MMP-9 in the tear is sufficiently high. This volume-dependency may be related to the capillary flow of solutes through the sample fleece. Among the different DED subtypes, Sjögren syndrome, ocular graft-versus-host disease, and Steven-Johnson syndrome cause severe tear depletion. Therefore, the MMP-9 in-situ test may yield negative or very weak positive results in these patients, despite a high concentration of MMP-9 in tears. Second, because anesthetic eye drops cannot be used when performing the MMP-9 in-situ immunoassay, care must be taken when tears are obtained from the conjunctiva because this process can induce reflex tears due to pain caused by contact between the cornea and the sample fleece. In this case, a false-positive result is possible since the band density is increased artificially. The volume-dependency of the assay means that careful attention must be paid to tear volume for precise interpretation. Also, as described above, identification of tear hyperosmolarity is the latest diagnostic method for diagnosis of dry eye; therefore, the authors aimed to investigate the effect of tear volume on measurement of tear osmolarity. Interestingly, in the case of the TearLab osmometer, the measured osmolarity was significantly higher when the sample volume decreased. We found that the measured osmolarity was higher than the average (20 mOsm/L) at both low (260 mOsm/L) and high (350 mOsm/L) concentrations. Therefore, as in the case of the MMP-9 in-situ test, careful interpretation of the results is required with respect to patients with reduced tear volume. As an extreme example, we found that when the MMP-9 in-situ test and tear osmolarity measurements were performed for patients with Sjögren syndrome, the tear osmolarity was significantly higher (due to the reduced tear volume), and the MMP-9 test result was likely to be completely negative. Furthermore, it is unlikely that the assay can generate quantitative results because tear sample volumes generated by every individual are not consistent^18,41^. In addition, when comparing the experimental results of the present study with the clinical results of previously reported papers, Lanza et al. found no difference in MMP-9 positivity between dry and non-dry eye groups. Furthermore, although there were no statistically significant differences, Schargus et al. found that the Schirmer test scores for the positive MMP-9 immunoassay group were higher than those of the negative patient group^42,43^. These results are consistent with the results herein, which show that tear volume plays an important role in positive results generated by the MMP-9 immunoassay.

A previous study examined the utility of the MMP-9 in-situ immunoassay for predicting responses to anti-inflammatory treatments in dry eye patients with confirmed ocular surface inflammation^44^. The authors used semi-quantitative evaluation of band density to distinguish the severity of inflammation. However, since it is not certain that the band density of the MMP-9 in-situ immunoassay increases in proportion to the severity of inflammation at the ocular surface, we examined the use of densitometry as a method for quantitative measurement of rhMMP-9. For sample volumes of 10 µl, the assay showed a linear concentration-dependent increase in band density. However, band density was saturated above 500 ng/ml at 20 µl. Full saturation of anti-analyte antibodies above a certain concentration at the test line is a common limitation of LFIAs. As mentioned earlier, since sample volumes vary due to the collecting method, examiner proficiency, or underlying disease, the band density will not reflect the exact concentration of MMP-9 in tears. In addition, our results suggested low reproducibility, indicating that even if the sample volume is controlled, the band density does not accurately reflect high or low levels of MMP-9. Nevertheless, a strong band suggests high MMP-9 concentrations or the existence of severe ocular surface inflammation in a patient; therefore, prompt anti-inflammatory treatment should ensue. Since LFIA-based test devices are recognized as appropriate strategies for screening disease, on/off (positive/negative) checks, or limited semi-quantitative evaluation, it is desirable to use this device only for these purposes^18,41^.

However, the InflammaDry kit instructions do not place much emphasis on incubation time. As shown in Fig. 4, we found that a constant increase in band density was observed after 20 min of testing. Therefore, to reduce false-positives, it may be appropriate not to exceed 20 min when the test is performed.

Drug interactions can also yield false-positive or false-negative results during tear MMP-9 testing. Although the manufacturer mentions that several types of eye drop can interfere with the test results, we tested a wide variety of drops. We found that 2% Isopto Carpine, Pazeo, and 1% Isopto Atropine strengthened the band density in the immunoassay. By contrast, Elazop, Lotemax, Cravit (0.5%), and Diquas reduced the band density. In relation to these effects, we hypothesize three possible mechanisms related to alteration of the colorimetric reaction in the MMP-9 immunoassay in the presence of various eye drops. First of all, individual eye drops have different pH values. At pH ~ 7.0, there is no significant effect on antigen–antibody reactions. However, low or high pH may inhibit the antigen–antibody reaction. Therefore, the colorimetric reaction may vary according to the pH of each eye drop. Second, the turbidity of each eye drop is different. For example, Elazop, Lotemax, and Ikervis are suspensions with a slightly milky color. This could interfere with development of the band in the immunoassay. However, the reason for the weak colorimetric reaction in the presence of most eye drops may be due to the viscosity of the drug itself^45^. If prolonged exposure of the ocular surface to eye drops is required, formulations may need to contain ingredients that increase viscosity. Patients with a variety of ophthalmic diseases often have dry eye symptoms or secondary DED caused by various drugs or surgery. Therefore, they take various medications or eye drops. This means that there is a high likelihood of medication-induced interference with the MMP-9 immunoassay. Therefore, a thorough history of medicines and dosing periods should be obtained prior to the test.

This study has some limitations. Nine ophthalmologists were asked to judge the results of MMP-9 in-situ assay to evaluate the detection limit of MMP-9 in-situ band density in a clinical setting. They read the results under standard conditions; however, we cannot rule out bias caused by not performing observer calibration or differences in inter-observer reliability. In addition, the present study used the active form of rhMMP-9, whereas actual tears contain both the preform and the active form of MMP-9; therefore, different results can be obtained under clinical conditions.

Due to its volume-dependency, the commercially available tear MMP-9 in-situ immunoassay InflammaDry provides limited quantitative information about MMP-9 concentrations in tears. The sample volume is the most critical factor for appropriate and accurate detection of MMP-9 above concentrations of 40 ng/ml, even in normal subjects. Therefore, clinicians should keep in mind that tear volume is crucial for accurate interpretation. Tear samples less than 10 µl may generate false-negative results, whereas tear samples more than 20 µl may generate false-positive results.
